# Improvement of renal function after human umbilical cord mesenchymal stem cell treatment on chronic renal failure and thoracic spinal cord entrapment: a case report

**DOI:** 10.1186/s13256-017-1489-7

**Published:** 2017-11-30

**Authors:** Ahmad Jabir Rahyussalim, Ifran Saleh, Tri Kurniawati, Andi Praja Wira Yudha Lutfi

**Affiliations:** 1 Department of Orthopaedic and Traumatology, Faculty of Medicine Universitas Indonesia / Cipto Mangunkusumo Hospital, Jakarta, Indonesia; 2 Stem Cell and Tissue Engineering Cluster, Faculty of Medicine Universitas Indonesia / Cipto Mangunkusumo Hospital, Jakarta, Indonesia

**Keywords:** Spinal cord entrapment, Chronic kidney failure, hUC-MSC

## Abstract

**Background:**

Chronic renal failure is an important clinical problem with significant socioeconomic impact worldwide. Thoracic spinal cord entrapment induced by a metabolic yield deposit in patients with renal failure results in intrusion of nervous tissue and consequently loss of motor and sensory function. Human umbilical cord mesenchymal stem cells are immune naïve and they are able to differentiate into other phenotypes, including the neural lineage. Over the past decade, advances in the field of regenerative medicine allowed development of cell therapies suitable for kidney repair. Mesenchymal stem cell studies in animal models of chronic renal failure have uncovered a unique potential of these cells for improving function and regenerating the damaged kidney.

**Case presentation:**

We report a case of a 62-year-old ethnic Indonesian woman previously diagnosed as having thoracic spinal cord entrapment with paraplegic condition and chronic renal failure on hemodialysis. She had diabetes mellitus that affected her kidneys and had chronic renal failure for 2 years, with creatinine level of 11 mg/dl, and no urinating since then. She was treated with human umbilical cord mesenchymal stem cell implantation protocol. This protocol consists of implantation of 16 million human umbilical cord mesenchymal stem cells intrathecally and 16 million human umbilical cord mesenchymal stem cells intravenously. Three weeks after first intrathecal and intravenous implantation she could move her toes and her kidney improved. Her creatinine level decreased to 9 mg/dl. Now after 8 months she can raise her legs and her creatinine level is 2 mg/dl with normal urinating.

**Conclusions:**

Human umbilical cord mesenchymal stem cell implantations led to significant improvement for spinal cord entrapment and kidney failure. The major histocompatibility in allogeneic implantation is an important issue to be addressed in the future.

## Background

The spinal cord can be injured due to the entrapment of the spinal canal. Spinal cord entrapment generally develops due to a mechanical insult. According to the degree of neural tissue damage, spinal cord entrapment results in various grades of irreversible disability of the motor and sensory functions. In particular, according to the level of the cord segment entrapment, the neurological dysfunction can extend to the whole body below the neck. In this case, even respiratory problems may develop due to the chest wall dysfunction resulting from high cervical cord intrusion. The need for more effective and safe treatment has led to a search for different therapeutic strategies that target the restorative stage over the acute narrow therapeutic window, such as cell-based therapies designed to regenerate damaged cells and simultaneously provide anti-inflammatory and/or neurotrophic factors to prevent the secondary neurodegeneration inherent in spinal cord injury (SCI) [1, 2]. Thoracic spinal cord entrapment may also be triggered by a deposit of metabolic factors in patients with renal failure.

**Table 1:** Preclinical studies using mesenchymal stem cells for the treatment of chronic kidney disease [3]

Disease	Source	Dose	Route	Mechanism of action	Side effects
Diabetic nephropathy	Mice bone marrow	0.5 × 10^6^ cells	Intravenous	Engraftment/direct effect	None
Diabetic nephropathy	Human bone marrow	2 × 10^6^ cells	Intracardiac	Engraftment/direct effect	None
Partial nephrectomy	Rat bone marrow	1 × 10^6^ cells	Intravenous	Paracrine effect	None
Chronic allograft nephropathy	Rat bone marrow	0.5 × 10^6^ cells	Intravenous	Immunomodulatory effect	None
Renal revascularization	Allogeneic swine adipose tissue	10 × 10^6^ cells	Intrarenal	Engraftment/direct effect/paracrine	None
Renal artery stenosis	Autologous swine adipose tissue	10 × 10^6^ cells	Intrarenal	Engraftment/direct effect/paracrine	None

Diabetes mellitus and hypertension represent major causes of chronic kidney disease (CKD) and initiation of dialysis in the USA. In addition, glomerular diseases, malnutrition, infectious diseases, and acute kidney intrusion can progress to end-stage renal disease (ESRD), contributing to the increased global burden of death associated with this condition. Current treatment modalities often fail to target the major underlying contributors for progression of renal disease. Chronic glomerular and tubulointerstitial fibrosis is a common pathway to ESRD, often associated with apoptosis, oxidative damage, and microvascular rarefaction. In fact, renal dysfunction is postulated to better correlate with the degree of tubulointerstitial than with glomerular damage. Of importance, the kidney possesses intrinsic regenerative capacity that allows the organ to recover after limited insults. Unfortunately, this regenerative potential is limited under chronic conditions and thus inefficient to prevent progressive glomerulosclerosis and tubulointerstitial fibrosis. Treatment strategies that boost cellular regeneration might therefore offer good alternatives for patients with CKD [3] (Table 1).

Mesenchymal stem cells (MSCs) are the main source of cell therapy because of their capability of differentiating into multiple cell types, including blood, adipose tissue, and connective tissue. These cells can easily grow *in vitro* and exhibit intriguing immunomodulatory properties, non-teratogenicity, and multi-potentiality with high genetic stability. MSCs can maintain regenerative capacity after cryopreservation, improve synaptic transmission, and promote neuronal networks. These properties make MSCs prime candidates for various therapeutic applications especially for nervous system repair. In recent years, experimental studies have uncovered the potential of MSCs to improve renal function in several models of CKD, and several clinical studies have indicated their safety and efficacy in CKD. However, a number of hurdles need to be addressed before clinical translation.

## Case presentation

We present a 62-year-old ethnic Indonesian woman previously diagnosed as having thoracic spinal cord entrapment with paraplegic condition for 6 months and chronic renal failure on hemodialysis for 2 years. She felt pain in her back and had difficulty in getting up from the floor. She could not move her legs, their power was 0/5, and there was no movement of her toes. She had no improvement in the neurologic deficit for 6 months. No sign of spinal shock was found in a physical examination during a neurological examination at her first visit. All modalities of sensation below her belly button were lost including proprioception. Urinary retention had to be catheterized, but she had no urine production for 2 years (please see information on kidney failure below). Defecation had to be helped by digital exploration. Her muscles were hypertonic with exaggerated jerk reflex and clonus. There was no history of significant injury to her back.

**Fig. 1:**
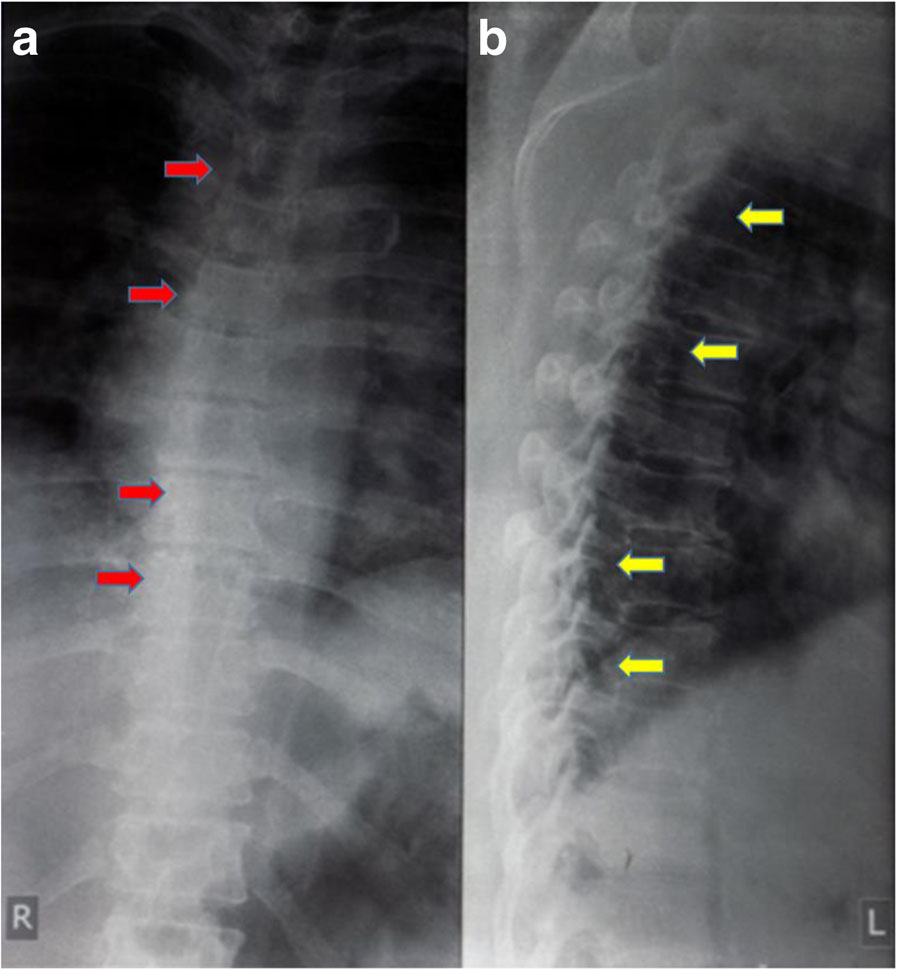
Plain radiograph of thoracic region, anteroposterior and lateral views. **a** Anteroposterior view shows spinal deformity and mass deposition process around the thoracic vertebrae (*red arrows*). **b** Lateral view shows degenerative process with mass deposition at some level of the spinal canal (*yellow arrows*)

**Fig. 2:**
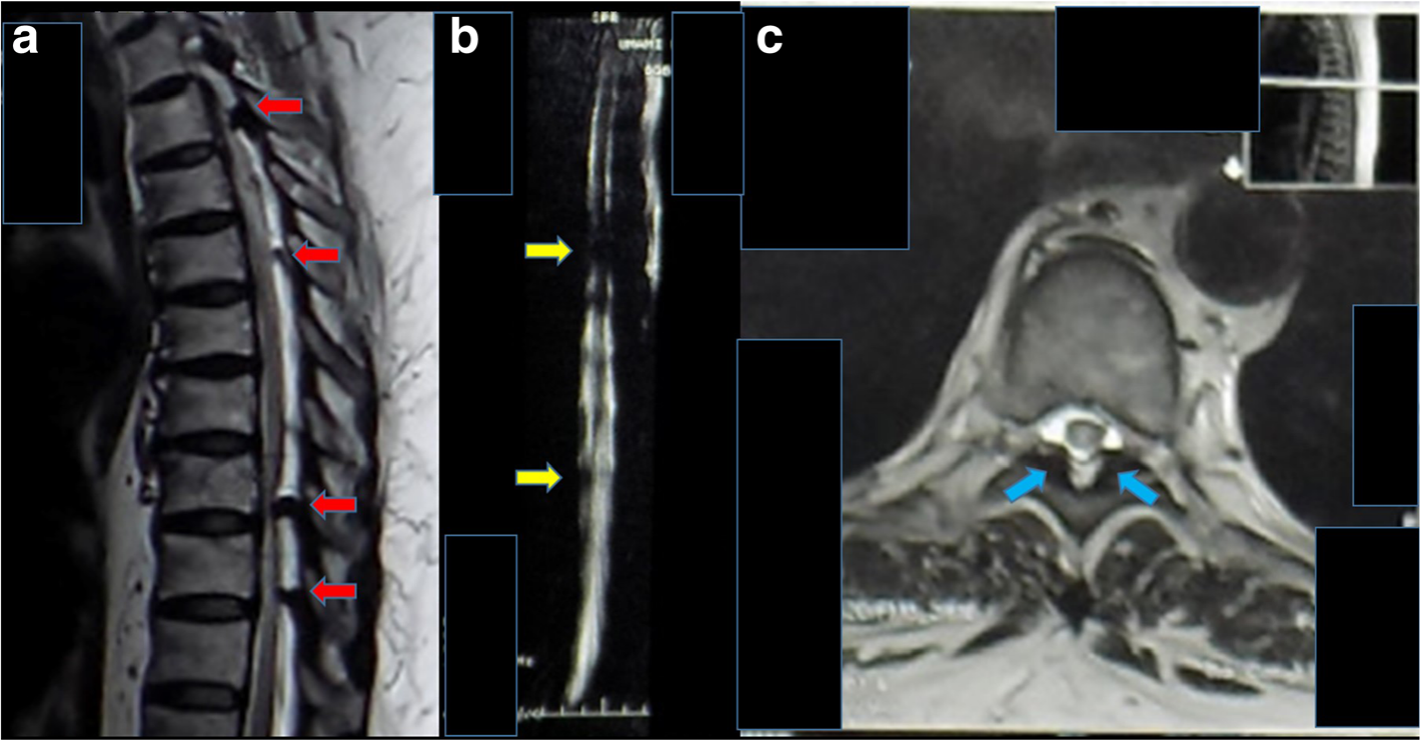
Sagittal and axial views of thoracic magnetic resonance imaging with T1-weighted and T2-weighted images. **a** Sagittal section of T2-weighted magnetic resonance imaging shows many compressions of spinal cord at levels T3, T5, T8, and T9 (*red arrows*). **b** Magnetic resonance imaging-myelography that shows spinal cord compression at levels T5 and T9 (*yellow arrows*). **c** Axial view of T5 vertebrae level shows the hypointense mass at left and right posterior parts (*blue arrows*). The mass compresses the canal

A chest and thoracic X-ray was taken and the result was normal (Fig. 1). T1-weighted magnetic resonance imaging showed a regular hypointense lesion in intradural location behind the bodies of Th8 and Th9 vertebrae predominantly on the left side. The lesion compressed the cord to the left. On T2-weighted magnetic resonance imaging the lesion was isointense relative to normal cord (Fig. 2).

The special note that we took here was that our patient had not urinated for the last 2 years before the paraplegic condition due to thoracic spinal cord entrapment. She had had diabetes mellitus for more than 10 years that already affected her kidneys. Two years before the incident, she had already been diagnosed as having ESRD. There is no history of diabetes mellitus, renal dysfunction, or paraplegic condition in her family. Her creatinine (Cr) level at that time was 11 mg/dl. She had to undergo hemodialysis three times a week.

We performed our protocol of human umbilical cord MSCs (hUC-MSCs) implantation and injection to treat this patient. The protocol consists of six cycles of hUC-MSCs intrathecal implantation and intravenous injection with time interval of 3 months. One cycle consists of implantation of 16 million hUC-MSCs performed three times and intravenous injection of 16 million hUC-MSCs performed three times. After the second cycle of hUC-MSC protocol, improvement was seen in both legs, and she could do extensions of both of her knees. Her sensory level was increased from 0 to 2, but the clonus was still there. Surprisingly, we also found that she had urinated for the first time in the last 2 years after the first cycle of hUC-MSC protocol, and her Cr level decreased significantly from 11 mg/dl to 2 mg/dl after the second cycle of hUC-MSC protocol. Fortunately, no signs of allergy were found in this patient; as we know, allergic effect is one of the most terrifying effects of intravenous injection of MSC.

## Discussion

**Fig. 3:**
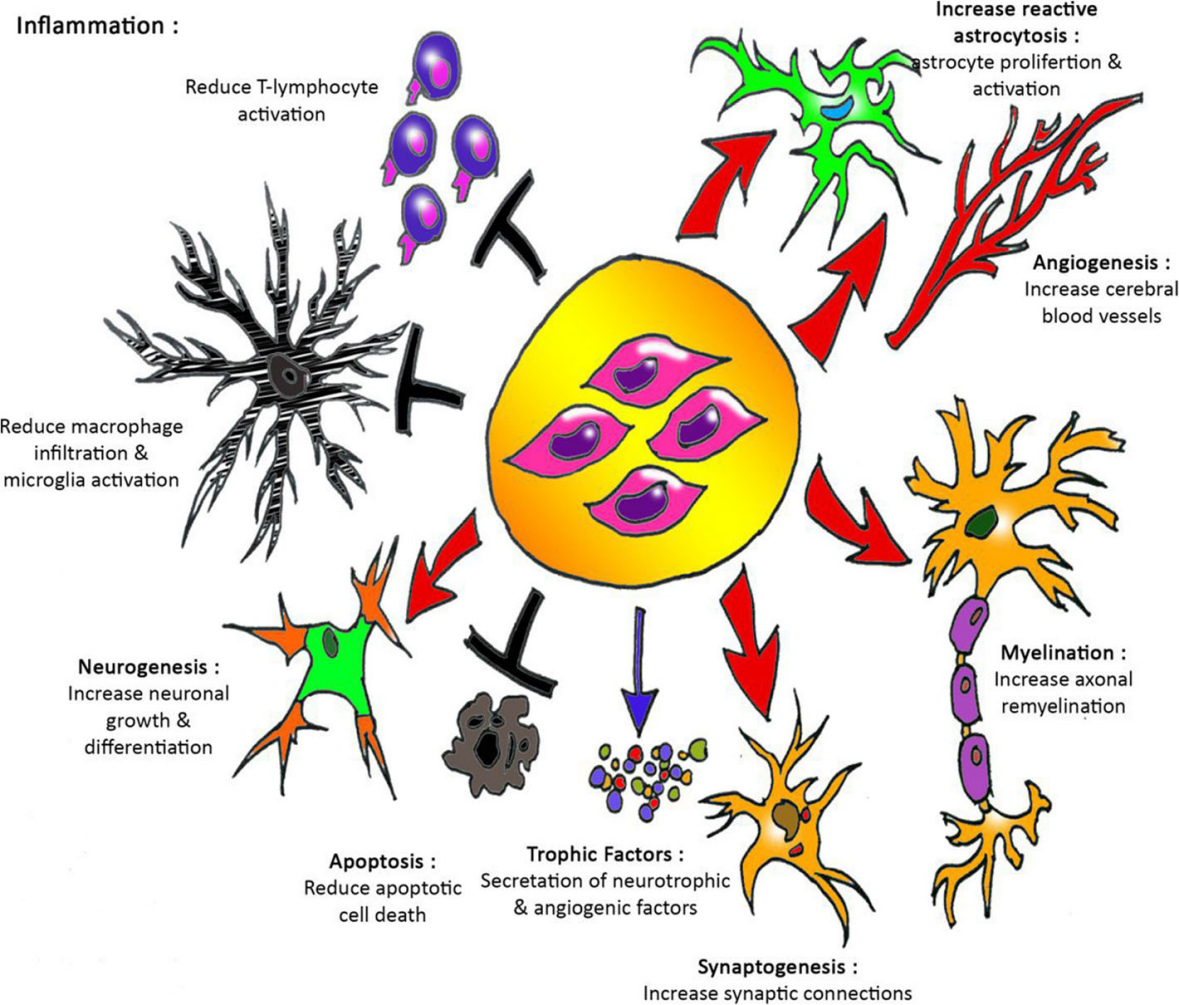
Potential neuroprotective and neurorestorative effects of mesenchymal stem cells. *MSCs* mesenchymal stem cells

A true stem cell is a totipotent cell. It can become any cell type present in an organism. Many consider the zygote to be the only true totipotent (stem) cell because it is able to differentiate into either a placenta cell or an embryonic cell. MSCs have a distinct self-renewal ability and differentiation potential. Whether they are cultured *in vitro* or *in vivo*, they can differentiate into osteoblasts, chondrocytes, adipocytes, myoblasts, and neuronal cells, suggesting their extensive clinical application potential. MSCs exist in a variety of tissue, such as bone marrow, periosteum, thymus, skin, adipose, muscle, umbilical cord, and umbilical cord blood. From the perspective of embryo development, the umbilical cord is the structure in which stem cells develop and migrate, and umbilical cord stromal cells have been found among populations of embryonic stem cells. The gelatinous connective tissue around the umbilical cord, called Wharton’s jelly, is a continuous skeleton formed by interwoven collagen and small fibers, and it contains a large number of myofibroblast-like mesenchymal cells [4]. Umbilical cord MSCs have a number of advantages which suggest that they might be an important source for allogeneic MSCs for cellular therapy as indicated by trends in MSC clinical trials worldwide. Compared to bone marrow MSCs, hUC-MSCs have many advantages, such as a wide variety of sources, easy acquisition, high proliferation ability, low immunogenicity, and fewer bioethics issues involved in their use. Therefore, hUC-MSCs are considered to be an ideal replacement for bone marrow MSCs. The optimization of the *in vitro* isolation and culture of hUC-MSCs and the examination of their biological properties are important prerequisites for their application (Fig. 3). Umbilical cords fall off after delivery and therefore constitute an easy access to cells, provide less possibilities of contamination, have no ethical concern, and are rich in MSCs. In addition, hUC-MSCs, unlike bone marrow MSCs, do not express tumor-associated fibroblast phenotypes and therefore have no opportunity to grow solid tumors [2, 4–6].

The damage from spinal cord intrusion is very complex, involving different types of cells. The microenvironment of the spinal cord changes considerably during the first few weeks after inflammation and scar formation and it is a very important event. After spinal cord intrusion, endogenous regenerative events occur, indicating that the spinal cord attempts to repair itself. Schwann cells, the myelinating and regeneration-promoting cells in the peripheral nervous system, migrate from spinal roots into the damaged tissue and myelinate spinal cord axons. The expression of regeneration-associated genes is increased in damaged neurons. There is a surge in proliferation of local adult stem cells and progenitor cells. However, axonal growth is thwarted by growth inhibitors present on oligodendrocyte myelin debris and on cells that form scar tissue. Also, the newborn stem cells and progenitor cells do not integrate functionally into the injured spinal cord tissue. Thus, the endogenous regenerative events that occur after intrusion fail to repair the spinal cord. A combination of therapies is needed at the appropriate time and on the correct target site. Studies in animals have shown that transplantation of stem cells can be an effective treatment strategy on spinal cord repair by replacing the nerve cells that have died, because of the intrusion, with differentiated neural stem cells. Transplantation of new supporting cells for myelin regeneration can be used to connect the injured axons and stimulate them for regrowth. Transplantation into the spinal cord intrusion site provides a protecting environment for cells at the disruption site to avoid further damage by releasing protective substances, such as growth factors, and it decreases toxins such as free radicals, and prevents spreading of the intrusion by suppressing the damaging inflammation that occurs after intrusion [7, 8].

**Fig. 4:**
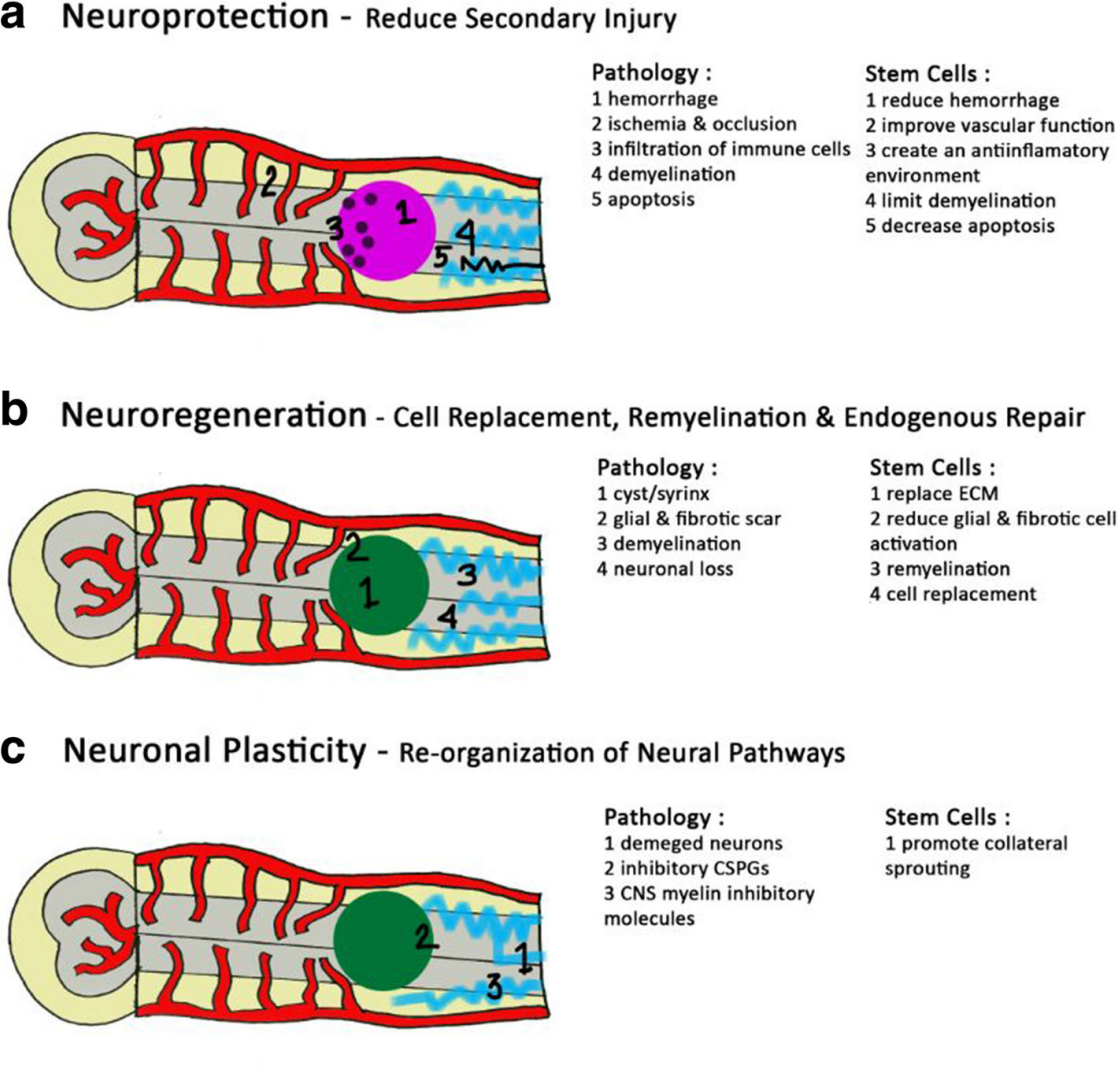
Stem cell therapies can result in neuroprotection, neuroregeneration, and/or enhance neuronal plasticity following spinal cord intrusion. **a** Neuroprotection refers to preservation and protection of neural tissue from secondary pathophysiology, including hemorrhage, ischemia as well as occlusion, infiltration of immune cells, demyelination, and apoptosis. Stem cells can be neuroprotective by reducing blood–spinal cord barrier disruption, improving vascular function, creating an anti-inflammatory environment, limiting demyelination, and decreasing apoptosis. **b** Neuroregenerative strategies aim to replace the damaged cells in the spinal cord by modifying the intrusion environment to either stimulate endogenous regeneration or exogenous cell transplantation. Stem cells can be neuroregenerative by providing an extracellular matrix scaffold within the cystic cavity, trophic support, remyelinating damaged axons, and cell replacement. **c** Damaged neurons, inhibitory chondroitin sulfate proteoglycans, and inhibitory components of central nervous system myelin restrict neuronal plasticity post-spinal cord injury. By promoting collateral sprouting, via trophic support, cell therapy can enhance the reorganization of neural pathways [11]. *BSCB* blood–spinal cord barrier, *CNS* central nervous system, *CSPGs* chondroitin sulfate proteoglycans, *ECM* extracellular matrix

The therapeutic potential of true stem/progenitor cells is still unknown. Other matters related to the use of stem/progenitor cells for spinal cord intrusion also need to be resolved before effective therapies can be developed. How can the cells be best obtained? Do they need to be differentiated *in vitro* before transplantation? How can survival of grafted stem/progenitor cells be improved and uncontrolled division and differentiation be prevented? How can functional integration of the transplanted cells be improved? The use of stem cells and the emergent field of regenerative medicine provide hope to patients with spinal cord intrusion but also raise a myriad of complex ethical issues. Stem cells have been of great interest to researchers because of the combination of two unique attributes. First, with specific treatment they are capable of prolonged self-renewal through division. Second, controlled physiological exposure may influence them to differentiate into specific cell lineages. Stem cells thus create hope for individuals with a wide range of conditions through the potential for repair and regeneration of diseased tissue. Cell therapies have neuroprotective as well as neuroregenerative potential in the context of spinal cord intrusion (Fig. 4). Therapeutic application of MSCs especially genetically modified MSCs opened up new research avenues in the treatment of spinal cord intrusion. However, cell therapy for spinal cord intrusion has several challenges including clinical application issues, ethical concerns, as well as transplantation methods, timing, and safety and efficacy of the transplanted cells. Also, complete and accurate understanding of the mechanisms of action and the behavior of stem cells in the pathological environment after transplantation is needed in order to determine the best time setting and the most efficient routes for cell delivery after the intrusion [2, 4, 9–11].

**Fig. 5:**
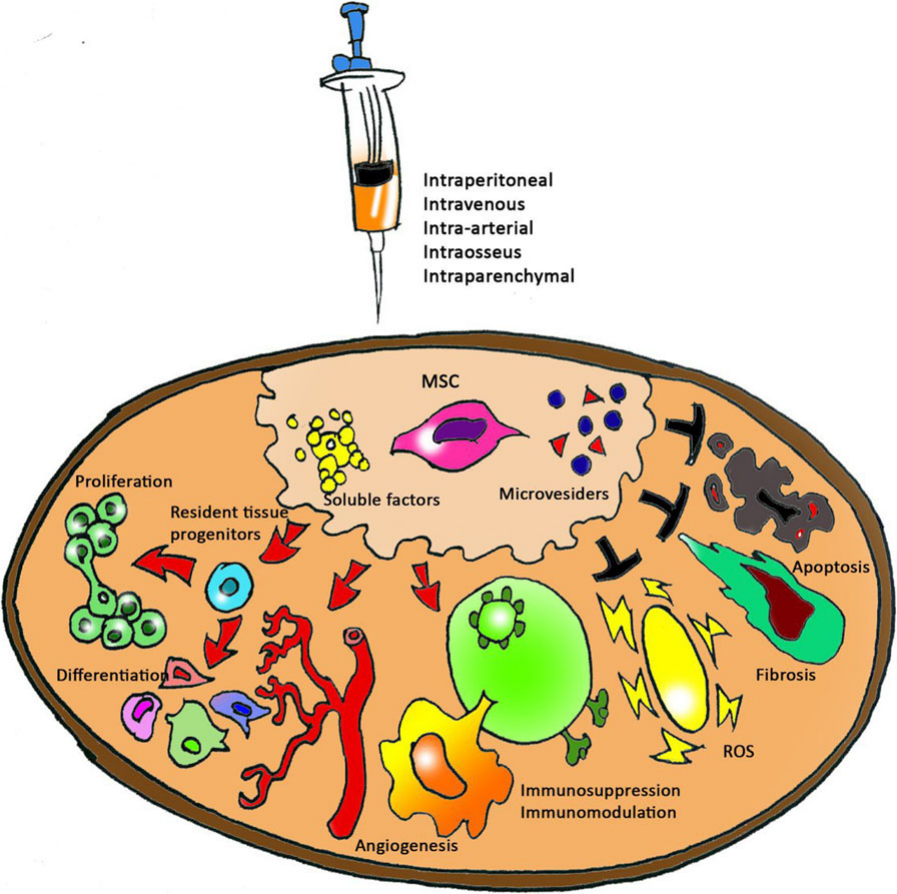
Properties of mesenchymal stem cell in kidney diseases. Mesenchymal stem cell, soluble factors, or microvesicles can be delivered to the kidney via the intraperitoneal, intra-arterial, intravenous, intraparenchymal, or intraosseous route. They exert a series of renoprotective and regenerative actions on the injured tissue through various paracrine mechanisms: antifibrotic and antiapoptotic, proangiogenic, proliferative and differentiative, antioxidative stress, and immunosuppression and immunomodulation of the immune system [6]. *MSC* mesenchymal stem cell, *ROS* reactive oxygen species, *Arrow* enhancement, *T-bar* reduction

The number of individuals affected with CKD is rising worldwide, mainly due to a remarkable increase in atherosclerosis and type 2 diabetes. CKD is characterized by reduced renal regenerative capacity. Several *in vivo* studies suggest beneficial regenerative effects of cell-based therapies in animal models of CKD (Fig. 5). First isolated and characterized by Friedenstein and colleagues in 1974, MSCs have emerged as ideal candidates for cell-based therapies for preservation of the human kidney. Of importance, MSCs secrete several growth factors and cytokines that modulate adjacent parenchymal cells, triggering tissue regeneration. The ability of MSCs to preserve renal structure and function has been demonstrated in experimental CKD, as their administration preserved renal function and attenuated renal injury in several rodent models of diabetic nephropathy, partial nephrectomy, and chronic allograft nephropathy. The therapeutic potential of MSCs is currently explained by their ability to regulate immune cells through paracrine interactions.

The key players in immune response and immunological diseases are T cells. It has been established that MSCs suppress T lymphocyte activation and proliferation and alter the T cell population towards an anti-inflammatory profile by increasing the number of regulatory T cells and repressing the differentiation of Th17 cells. Over the past few years, MSCs have been successfully applied in experimental models of CKD such as diabetes, hypertension, and chronic allograft nephropathy. For example, a single intravenous dose of MSCs resulted in beta-pancreatic islet regeneration, prevented renal damage in streptozotocin-induced type 1 diabetes in C57BL/6 mice, and decreased hyperglycemia and glycosuria that persisted for 2 months after injection. Furthermore, MSC-treated diabetic mice showed histologically normal glomeruli and albuminuria fell. Although the authors did not assess cellular mechanisms associated with MSC therapeutic effects, the long-lasting persistence of injected MSCs may suggest a direct effect to elicit kidney regeneration.

Kilpinen *et al*. [12] showed that hUC-MSCs secrete constitutively extracellular membrane vesicles (MVctrl) capable of significantly attenuating ischemia/reperfusion kidney injury in rats. The promising results obtained from numerous *in vitro* and *in vivo* experiments using MSCs created great enthusiasm in the scientific community, offering new possibilities of cell-based therapies for a wide range of diseases. Over the past several years, the discrepancy between the number of wait-listed patients and the number of kidneys from brain-dead donors has been increasing steadily, leading to a shortage of organs and resulting in an extension of the criteria for kidney donors, including non-heart-beating donors (NHBD). However, kidneys from NHBD are damaged during the period of warm ischemia associated with cardiac death. Recent studies suggested the possibility of potentiating the intrinsic reparative capacity of MSCs through preconditioning or genetic modification. Once fully tested, enhanced MSCs could become an important new tool for current as well as unexplored therapeutic fields [6].

## Conclusions

Human umbilical cord stem cells have been shown to have important neuroprotective and neuroregenerative effects following spinal cord entrapment and simultaneously work for improvement of kidney failure.

## Abbreviations

CKD: Chronic kidney disease
Cr: Creatinine
ESRD: End-stage renal disease
hUC-MSC: Human umbilical cord mesenchymal stem cells
MSCs: Mesenchymal stem cells
NHBDs: Non-heart-beating donors
