# Correction: Improvement of renal function after human umbilical cord mesenchymal stem cell treatment on chronic renal failure and thoracic spinal cord entrapment: a case report

**DOI:** 10.1186/s13256-023-03939-5

**Published:** 2023-05-09

**Authors:** Ahmad Jabir Rahyussalim, Ifran Saleh, Tri Kurniawati, Andi Praja Wira Yudha Luthfi

**Affiliations:** 1 Department of Orthopaedic and Traumatology, Faculty of Medicine Universitas Indonesia/Cipto Mangunkusumo Hospital, Jakarta, Indonesia; 2 Stem Cell and Tissue Engineering Cluster, Faculty of Medicine Universitas Indonesia/Cipto Mangunkusumo Hospital, Jakarta, Indonesia

**Correction: Journal of Medical Case Reports (2017) 11:334** 10.1186/s13256-017-1489-7

Following publication of the original article [1], the authors identified an error in the author name of Andi Praja Wira Yudha Luthfi.

The incorrect author name is: Andi Praja Wira Yudha Lutfi

The correct author name is: Andi Praja Wira Yudha Luthfi

The author group has been updated above and the original article [1] has been corrected.
